# Adrenal Mass Evaluation: Suspicious Radiological Signs of Malignancy

**DOI:** 10.3390/cancers17050849

**Published:** 2025-02-28

**Authors:** Giulia Grazzini, Silvia Pradella, Federica De Litteris, Antonio Galluzzo, Matilde Anichini, Francesca Treballi, Eleonora Bicci, Vittorio Miele

**Affiliations:** 1 Department of Radiology, Careggi University Hospital, 50134 Florence, Italy; pradella3@yahoo.it (S.P.); federica.delitteris@gmail.com (F.D.L.); antonio.galluzzo@unifi.it (A.G.); matyanichini@gmail.com (M.A.); francescatreballi@gmail.com (F.T.); eleonora.bicci92@gmail.com (E.B.); vmiele@sirm.org (V.M.); 2 Department of Experimental and Clinical Biomedical Sciences “Mario Serio”, University of Florence, 50134 Florence, Italy

**Keywords:** adrenal gland, incidentaloma, computed tomography, magnetic resonance, adrenocortical carcinoma, pheochromocytoma, texture analysis, radiomic

## Abstract

An adrenal mass discovered incidentally during imaging for unrelated clinical reasons is termed an “adrenal incidentaloma” (AI). AIs can be categorized as primary or metastatic, functioning or non-functioning, and benign or malignant. The primary goal of radiological evaluation is to exclude malignancy by differentiating between benign and malignant lesions. Most AIs are benign, with adenomas and macronodular bilateral adrenal hyperplasia being the most common types. Less common benign lesions include myelolipomas, pheochromocytomas, cysts, and hematomas. Malignant adrenal masses account for less than 10% of cases and often include metastases from other cancers or primary adrenal diseases, such as adrenocortical carcinoma and pheochromocytoma. Computed Tomography (CT) remains the gold standard for diagnosing adrenal incidentalomas, while Magnetic Resonance Imaging (MRI) and Positron Emission Tomography (PET) are utilized for indeterminate cases. Additionally, innovative imaging techniques such as texture analysis are gaining importance, as they can assess quantitative parameters that are not visible to the human eye. This review aims to provide an updated overview of malignant adrenal lesions on CT and MRI, emphasizing key imaging features suspicious for malignancy to aid in distinguishing between benign and malignant lesions. Furthermore, it highlights the growing role of radiomics as a supportive tool for radiologists.

## Simple Summary

Adrenal incidentalomas (AI) are masses incidentally discovered during imaging performed for unrelated clinical reasons. The primary goal of radiological evaluation is to exclude malignancy by distinguishing between benign and malignant lesions. Computed Tomography (CT) is the gold standard for diagnosis, while MRI and PET are used for indeterminate cases. Advanced imaging techniques like Radiomic are gaining importance for assessing AIs. The review aims to provide an updated overview of malignant adrenal lesions on CT and MRI, emphasizing radiological signs of malignancy and the growing role of radiomics as a support tool for radiologists.

## 1. Introduction

An adrenal mass larger than 1 cm, incidentally identified during imaging performed for unrelated clinical reasons, is commonly referred to as an “adrenal incidentaloma” (AI) [1,2]. The prevalence of AIs increases with age, ranging from approximately 3% at age 50 to 10% in the elderly [3]. AIs can be classified as primary or metastatic, functioning or non-functioning, and benign or malignant lesions [4].

The identification of AIs through Computed Tomography (CT) and Magnetic Resonance Imaging (MRI) is becoming increasingly common [5,6]. Therefore, it is essential for radiologists to be skilled in evaluating these lesions [7,8]. The primary goal of initial radiological assessment is to exclude malignancy and distinguish between benign and malignant lesions [9,10,11]. Moreover, identifying the type of malignant lesion is crucial, as treatment varies depending on the nature of the mass [3,12].

**Figure 1:**
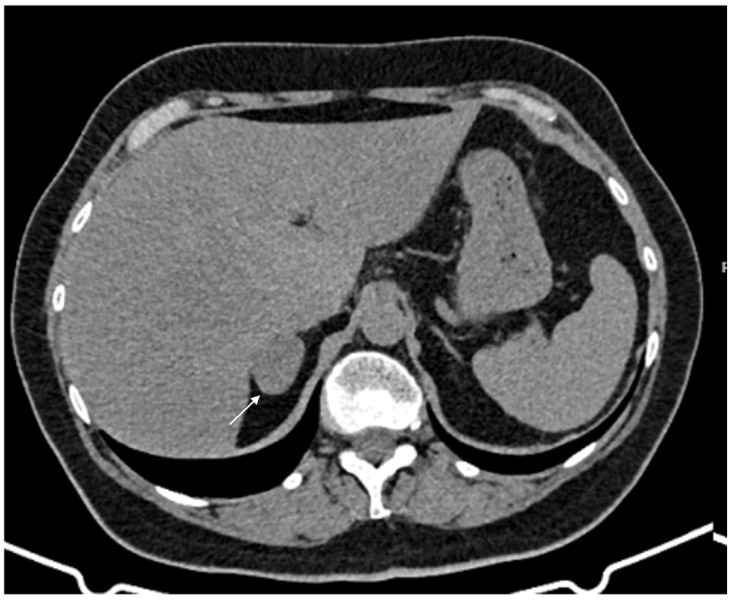
60-year-old woman with adrenal adenoma. Accidental CT finding of a right adrenal mass (arrow) of approximately 3 cm, homogeneous with clear margins and density values lower than 10 HU.

**Figure 2:**
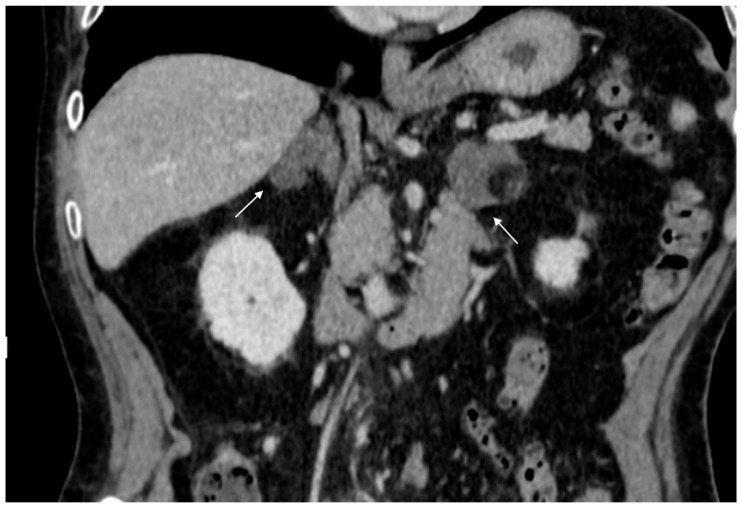
74-year-old male patient. Following an MRI of the digestive tract incidental finding of bilateral adrenal secondary hyperplasia. Both enlarged limbs adrenal glands > 10 mm thick with multiple nodularity hypodense on non-contrast CT (arrows).

When an incidentaloma is detected, it is usually benign. The most frequent benign cortical tumor among AIs is adenoma (AA), which can be further classified into lipid-rich adenomas (LRA) or lipid-poor adenomas (LPA). Macronodular bilateral adrenal hyperplasia is also common, comprising 80–85% of benign cases (Figure 1 and Figure 2). Other rarer benign lesions include myelolipomas (3–6%), pheochromocytomas (1–5%), cysts and pseudocysts (1%), hemorrhages and hematomas (<1%), ganglioneuromas (1%), lymphangiomas, schwannomas (<1%), and vascular malformations [13,14,15,16,17,18]. Malignant adrenal masses account for less than 10% of cases [19].

**Figure 3:**
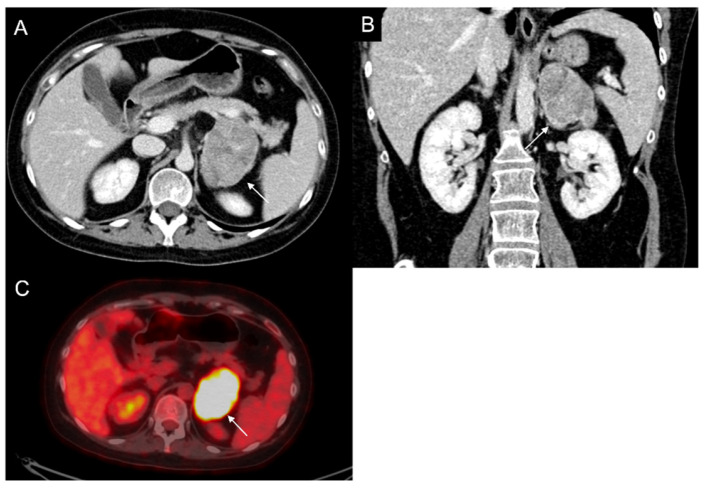
60-year-old woman with history of breast cancer and adrenal metastasis. Left adrenal mass with inhomogeneous enhancement (arrow) on CT portal phase in axial (**A**) and coronal (**B**) planes, and positive PET (**C**).

**Figure 4:**
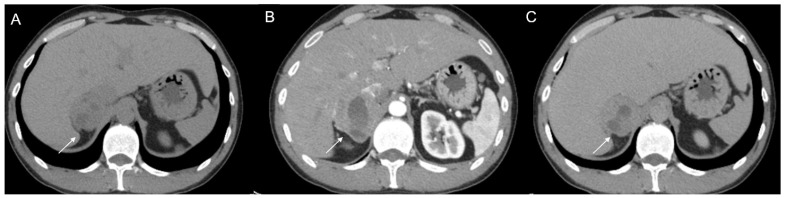
36-year-old man with history of night sweats, hypertension, and tachycardia due to pheochromocytoma (arrow). Non-contrast CT shows an inhomogeneous right adrenal mass > 20 HU (**A**). In the arterial phase (**B**), the mass shows intense enhancement of the solid components with persistent enhancement. In the late phase (**C**), absolute contrast medium washout is <50%.

The most common malignant adrenal lesions are metastases, typically originating from lung, breast, stomach, liver, kidney, pancreas, and colon cancers, as well as melanomas and malignant lymphomas (Figure 3) [20]. Alternatively, primary adrenal diseases such as pheochromocytomas or adrenocortical carcinomas (ACC) may be present (Figure 4) [9]. ACC is a rare and aggressive malignant tumor of the adrenal cortex, with a poor prognosis and an incidence of approximately one case per million people per year. The most common clinical manifestation of ACC is cortisol overproduction, observed in up to 40% of cases [21]. Pheochromocytomas are rare, typically benign tumors (90%), though a small percentage may be malignant [22,23]. They are associated with catecholamine production and are evaluated by measuring metanephrine and normetanephrine levels [1,24]. Less common malignant adrenal lesions include lymphomas and neuroblastomas, one of the most common abdominal tumors in children [7,25,26].

In more than 15% of cases, adrenal incidentalomas are bilateral [27]. Of all incidentally discovered adrenal lesions, approximately 10% are functional and may secrete steroids, mineralocorticoids, sex hormones, or catecholamines [28]. These lesions exhibit a wide range of clinical presentations. They may be asymptomatic or cause symptoms such as hypertension, weight gain, blood glucose changes, menstrual irregularities, and hirsutism in women, typical of cortisol-secreting adenomas [29].

Accurate diagnosis and characterization of these lesions are critical, given the vast differences in treatment approaches. Initial evaluations should determine whether the mass is functional or non-functional through laboratory analyses. Ruling out malignancy is mandatory. Although biopsy with histological analysis is the gold standard for characterization, its role is limited, while radiological imaging is becoming increasingly significant [30]. Multiphasic CT is the gold standard for diagnosing and characterizing adrenal masses [31]. Dual-energy CT, which provides virtual measurements of attenuation and iodine density without contrast, is also being studied [32,33]. MRI is a valuable alternative for indeterminate masses or when iodinated contrast cannot be administered [31]. Fluorine-18-2-fluoro-2-deoxy-D-glucose (FDG) positron emission tomography (PET) can differentiate benign from malignant adrenal lesions, with the adrenal-to-liver standardized uptake value ratio being a key discriminative factor [32]. Ultrasonography (US) and contrast-enhanced ultrasound (CEUS) are also being evaluated for their potential to differentiate benign from malignant adrenal masses [31].

CT and/or MRI images, however, may not always characterize all adrenal incidentalomas, particularly those with heterogeneity caused by calcifications, hemorrhage, or necrosis. Innovative imaging techniques, such as texture analysis, are gaining importance [34]. Radiomics and texture analysis allow for the assessment of tissue heterogeneity by applying regions of interest (ROIs) to CT and MRI images. By analyzing the tissue structure, these methods extract quantitative features, including first-, second-, and higher-order characteristics, that are not visible to the human eye [35].

This review aims to provide an updated overview of malignant adrenal lesions on CT and MRI, emphasizing the growing importance of radiomics as a valuable tool for radiologists. It also highlights imaging features suspicious for malignancy to aid in distinguishing between benign and malignant lesions.

## 2. Pearls and Pitfalls

Several parameters need to be considered to assess the normality of the adrenal gland, and it is critical to be careful not to fall into some pitfalls that could cause adrenal lesions mischaracterization, such as the presence of pseudo-lesions [36].

Evaluating the shape and the size of adrenal glands is essential to define its normality or abnormality. However, the adrenals have a range of morphological variability that makes their assessment difficult. The most common shape of adrenal glands was found to be the inverted Y shape, followed by the V and triangular shapes. John et al., in their study on 586 adults without adrenal disease, found the cumulative thickness of normal adrenal glands was 15.6 ± 3.7 mm and 18.4 ± 3.8 mm on the right and left sides, respectively, with significant differences between men and women [37].

**Figure 5:**
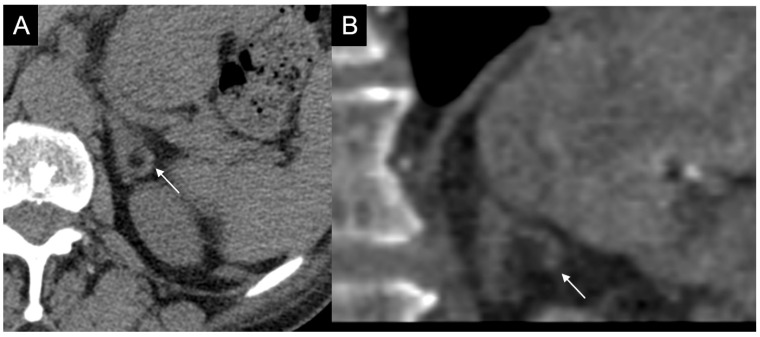
Doubtful hypodense nodule (arrow) inside the left adrenal gland on basal CT (**A**); in coronal reconstructions the adrenal gland has normal morphology (**B**).

Evaluating images CT or MR on multiple planes helps to avoid falling into pitfalls. A review by Khaled M. Elsayes et al. shows that in some cases, a normal adrenal gland may be mistakenly identified as a pathological adrenal nodule. This misidentification may be attributed to the appearance of the gland when viewed in a single plane, most commonly the axial plane [36]. Because of normal variations in the shape, position, and orientation of the adrenal glands, a horizontally oriented adrenal gland limb may simulate an adrenal nodule in the axial plane. However, by evaluating multiplanar reformatted images (MPR) in the coronal and sagittal planes, it can be understood that the gland is normal. Similarly, a vertically oriented limb can be easily characterized in the axial plane but may simulate an adrenal nodule in the coronal plane. Therefore, it is always useful to use MPR on CT or alternative imaging planes on MRI if suspicious nodules are detected (Figure 5) [36].

Other pitfalls can result from nonadrenal lesions with an inconspicuous cleavage plane [38,39]. Gastric diverticula and exophytic gastric tumors, such as gastrointestinal stromal tumors, can mimic adrenal lesions if they extend into the left suprarenal space; but also, renal pathologic conditions, pancreatic tail pathologic conditions, and exophytic hepatic. Other images that can deceive are tortuous, dilated, or aneurysmal vessels because they may seem adrenal lesions, especially splenic varices and splenic artery pseudoaneurysms [40]. A common problem is the presence of accessory spleens, particularly wandering spleens, which can simulate adrenal masses. Usually, accessory spleen measure less than 3 cm, whereas adrenal carcinoma can reach even larger sizes. However, in more complicated cases it may be useful to rely on magnetic resonance angiography (MRA) and damaged red blood cell (RBC) scintigraphy to differentiate them [41].

Moreover, since adrenal masses are generally incidental, the CT/MRI acquisition protocol as well as the report are often incomplete. In this setting, using a structured report could help avoid errors and incompleteness [42].

## 3. Guidelines for the Management of Adrenal Incidentaloma

According to the American College of Radiology (ACR) Incidental Findings Committee, when adrenal incidentalomas (AIs) are discovered on imaging, three key factors should be considered: the presence of symptoms suggestive of a hyperfunctioning mass, a history of malignancy that increases suspicion for metastasis (with an estimated probability of 26–36%), and the availability of prior abdominal imaging to assess for growth over time. While most adrenal lesions only require monitoring, certain cases may necessitate percutaneous biopsy, dual-energy CT, or comprehensive PET/CT scans [43].

The updated 2021 ACR white paper provides specific recommendations for managing AIs. For patients with an indeterminate adrenal mass smaller than 1 cm identified on initial imaging and no history of malignancy, neither CT nor MRI is typically warranted [44]. In contrast, for patients without a history of malignancy and an AI measuring between 1 and 4 cm that lacks diagnostic benign imaging features, follow-up imaging at 6 to 12 months is recommended to assess for stability in size. In this context, FDG-PET/CT is not advised for initial evaluation, and image-guided biopsy is deemed inappropriate. If an indeterminate adrenal mass is larger than or equal to 4 cm on initial imaging and there is no prior history of malignancy, surgical resection is recommended due to the high likelihood of adrenocortical carcinoma. For patients with a history of malignancy and an indeterminate adrenal mass less than 4 cm, adrenal-specific imaging should be conducted. If diagnostic results are inconclusive, further evaluation with adrenal biopsy or FDG-PET/CT is appropriate. Similarly, FDG-PET/CT or biopsy should be prioritized for patients with an indeterminate adrenal mass larger than 4 cm and a history of malignancy [44].

**Figure 6:**
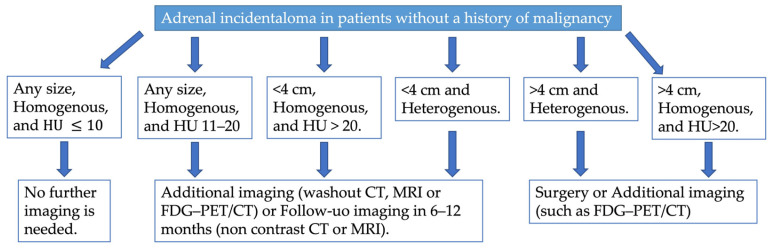
Proposed diagnostic flow charts for adrenal incidentaloma in patients without a history of extra-adrenal malignancy.

In 2023, the European Society of Endocrinology updated its guidelines for managing adrenal incidentalomas. The guidelines emphasize the importance of multidisciplinary expert team discussions involving an experienced endocrinologist, radiologist, and surgeon for some patients with adrenal incidentalomas [19]. Upon discovery of an adrenal mass, they recommend assessing malignancy risk and evaluating for functionality through clinical examination for symptoms or signs of hormone excess. Non-contrast CT is the recommended initial imaging modality for evaluating an AI (Figure 6). Homogeneous lesions with Hounsfield units (HU) ≤ 10 are highly likely to be benign and require no further imaging, regardless of size. However, additional imaging—such as CT with delayed contrast washout, FDG-PET/CT, or MRI with chemical shift—is suggested for patients with AIs larger than 4 cm, homogeneous lesions with HU between 11–20, or heterogeneous lesions smaller than 4 cm with HU > 20 (Figure 6). Multidisciplinary discussions are recommended for these patients to determine further steps. Lesions larger than 4 cm that are inhomogeneous or have HU > 20 are considered to carry a high enough risk of malignancy to warrant surgical intervention (Figure 6). Patients with adrenal lesions displaying clear benign imaging features require no follow-up. For indeterminate adrenal masses that are not surgically removed, repeat non-contrast CT or MRI is recommended 6–12 months after the initial study to check for significant growth. Surgical resection is suggested if the lesion increases in maximum diameter by more than 20% (or by at least 5 mm) during this period. Alternatively, further imaging after another 6–12 months may be considered [19].

For patients with bilateral or multiple adrenal lesions, each adrenal mass should be individually assessed at the time of initial detection using the same imaging protocol applied to unilateral masses. This evaluation aims to determine whether each nodule is benign or malignant [19].

**Figure 7:**
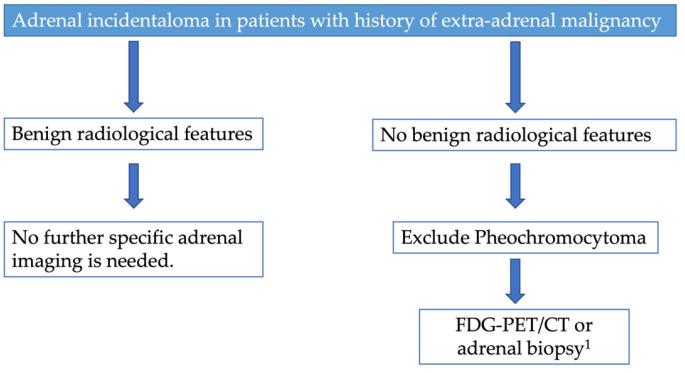
Proposed diagnostic flow charts for adrenal incidentaloma in patients with a history of malignancy. ^1^ Adrenal biopsy in selected patients, particularly if knowledge of the histology can influence management decisions.

In patients with a history of extra-adrenal malignancy, adrenal lesions deemed benign on non-contrast CT do not require specific follow-up imaging (Figure 7) [19]. On the other hand, adrenal lesions without benign diagnostic features on CT/RM or documented stability in patients with a history of malignancy require further evaluation such as FDG-PET/CT or biopsy (Figure 7). As a first step, the European Society of Endocrinology recommends excluding pheochromocytoma in these patients measuring plasma or urinary metanephrines [19]. Moreover, 18-Fdihydroxylphenylaalanine (DOPA), Ga-68-DOTATATE, or I-123 metaiodobenzylguanidine (MIBG) can be utilized to improve detection of pheochromocytoma, as recommended by ACR [44]. In patients with indeterminate adrenal mass and a history of extra-adrenal malignancy, both the ACR and the European Society of Endocrinology recommend FDG-PET/CT that showed an excellent sensitivity (between 93% and 100%) in distinguishing between a benign and a malignant mass [19,44]. In patients with a history of extra-adrenal malignancy, an adrenal mass showing high FDG uptake on PET-CT is likely to be a metastasis, although some malignant lesions may be FDG-negative, especially renal cancer [19]. Moreover, both ACR and the European Society of Endocrinology agree that biopsy should only be performed in selected cases. In particular, the adrenal biopsy should only be considered after exclusion of a pheochromocytoma and if knowledge of the histology can influence management decisions [19]. Adrenal biopsy can have a non-diagnosis rate of 4–19% while, if the biopsy sample is sufficient, the accuracy of the biopsy is between 96% and 100% for malignant lesions [44]. Moreover, adrenal biopsy may present complications such as bleeding, pneumothorax, infection, and tumor dissemination with rates ranging from 8% to 12% [44].

## 4. Suspicious CT Signs

Unenhanced CT is the primary imaging modality for evaluating adrenal masses and measuring Hounsfield units (HU), which reflect the attenuation value on non-contrast CT scans. A key benign imaging feature is a density value of ≤10 HU, which is highly specific for lipid-rich lesions and indicates a strong likelihood of adenomas [45]. In fact, the European Society of Endocrinology concluded that an adrenal lesion “with a density of ≤10 HU are virtually never malignant” because CT density > 10 HU showed 100% of sensitivity for detecting a malignant lesion in many studies. However, the specificity was lower, ranging between 52.9 and 71.8%, which means that many benign adrenal lesions show HU > 10. If the cutoff for the benign/malignant distinction is set to 20 HU, sensitivity remains high (97%) while specificity improves (range between 65.8 and 83.4%) [19].

When performing non-enhanced adrenal CT, it is essential to use an appropriate tube voltage, typically around 120 kVp, as altering the tube voltage impacts soft-tissue densitometry [40]. Additionally, the region of interest (ROI) should cover one-half to two-thirds of the adrenal mass and be placed over a homogeneous area, avoiding necrotic regions [46].

**Figure 8:**
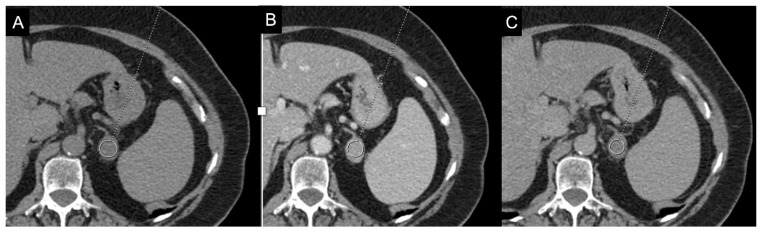
79-year-old female patient with left adrenal lesion detected at abdominal ultrasound performed for abdominal pain. Homogeneous left adrenal mass with density on non-contrast CT (**A**) less than 10 HU (6 HU, SD4 HU) and washout (>60%) in the venous (**B**) and late post-contrast phases (**C**), compatible with adenoma.

For lesions with a basal density value > 10 HU, supplemental imaging with intravenous contrast is useful to further evaluate the gland, particularly in the delayed phase [7]. Lipid-poor benign adrenal lesions (LPA) with attenuation values > 10 HU are not uncommon (10–40%), making differentiation from malignant lesions challenging [47]. Attenuation values > 10 HU on unenhanced CT, combined with rapid enhancement and slow washout on contrast imaging, raise the suspicion of malignancy. For this reason, delayed CT images are recommended to calculate the absolute percentage washout (APW) and relative percentage washout (RPW) [48]. Caoili et al. demonstrated that both LP and LR adenomas exhibit an APW ≥ 60% and an RPW ≥ 40%, pointing out that they can be distinguished from nonadenomas on the basis of these percentages [49]. Liu et al., in their study on 116 adrenal masses, found that the RPW provides the best accuracy (86%), with 85% sensitivity and 90% specificity, while the APW criterion yields an accuracy of 81%, 85% sensitivity, and 69% specificity (Figure 8) [50]. However, exceptions exist: Adrenal metastases from hypervascular primary tumors (e.g., renal cell carcinoma, hepatocellular carcinoma) and adrenal cortical carcinoma (ACC) can show APW and RPW values within the adenoma range. Furthermore, pheochromocytomas, which usually display slow washout, can occasionally mimic adenomas with rapid washout [51]. In fact, as demonstrated by the methanalaysis of Dinnes et al., there is very limited data on the accuracy of these cutoffs in adrenal incidentalomas [52]. A recent study demonstrated that the cutoffs of 60% for APW and 40% for RPW misclassified 35.9% (using absolute washout) and 35.2% (relative washout) of the adrenal masses with unenhanced attenuation values > 10 HU. They suggested a cutoff of 58% for RPW since using this threshold all malignant tumors were correctly identified [53]. However, this suggested cutoff requires confirmation from other studies. Moreover, there is some uncertainty regarding the optimal timing for delayed imaging. However, the latest guidelines of European Society of Endocrinology suggest that a delay of at least 10 min is necessary, with 15 min being more effective [19].

For adrenal masses with increased attenuation on non-enhanced CT (>45 HU) that remain indeterminate after evaluating morphology and washout, FDG PET/CT is preferable over additional CT or MRI, especially in oncologic patients [40].

**Figure 9:**
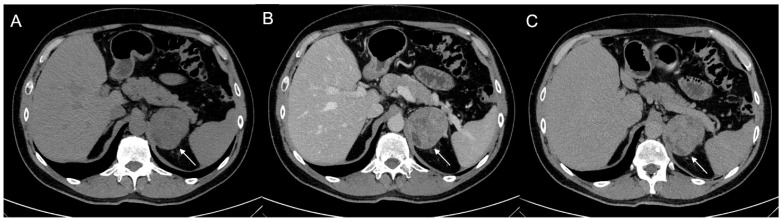
Voluminous lesion (arrow) of the left adrenal gland measuring 60 × 55 mm with density value > 20 HU on basal CT (**A**) and inhomogeneous enhancement on venous (**B**) and late post-contrast phases (**C**) due to the presence of necrotic and cystic areas. Histological diagnosis of Adrenocortical carcinoma.

**Figure 10:**
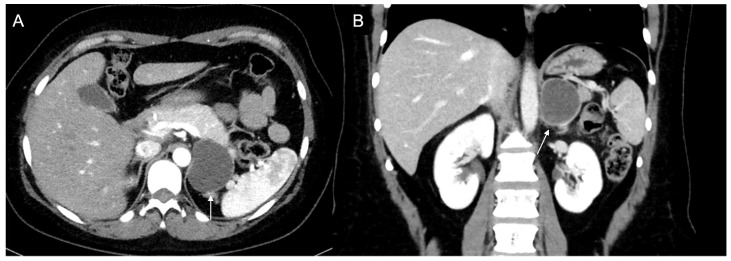
Man 60 years old with left adrenal incidentaloma. Hypodense mass (arrow) without contrast enhancement on CT (**A**,**B**), typically benign, stable in follow-up.

Another critical CT criterion for malignancy is lesion size [54]. Adrenal incidentalomas with a short axis < 1 cm are usually benign and do not require further evaluation. However, larger lesions have a higher likelihood of malignancy. Adrenal masses > 4 cm carry a 10% probability of malignancy, and 95% of ACCs exceed 5 cm in size [55]. A 2020 study by Bancos et al., involving adult patients (age ≥ 18 years), found that 98% of ACCs were >4 cm in size and 99% exhibited unenhanced CT attenuation values > 20 HU (Figure 9) [56]. According to Chatzellis and Kaltsas, CT density values ≤ 10 HU outperform size in differentiating benign from malignant masses. For instance, a homogeneous 5 cm adrenal mass with a CT attenuation value ≤ 10 HU has a near-zero risk of malignancy (Figure 10) [57].

Lesion stability or growth over time is another critical factor. Lesions that increase in size are more suspicious for malignancy than those that remain stable for over a year [44]. If an adrenal lesion enlarges during follow-up, metastasis is the most likely diagnosis to exclude, as metastases are the most frequent malignant adrenal lesions. Metastases often exhibit high unenhanced CT attenuation values (>20 HU), strong portal venous enhancement, and slower washout compared to adenomas [7]. They are often bilateral, with irregular edges and central necrosis, while calcifications are rare [58].

Malignant adrenal lesions, in general, are typically heterogeneous, displaying necrosis, variable intracellular lipid content, hemorrhage, and calcifications [9]. A retrospective study by Altinmakas et al. evaluated 47 pathologically confirmed adrenal masses (19 adenomas and 28 pheochromocytomas) using adrenal CT. They found that many smaller pheochromocytomas exhibited adenoma-like washout patterns on CT. Features most suggestive of pheochromocytomas include necrosis, strong and rapid heterogeneous enhancement, and higher attenuation [47]. Typically, pheochromocytomas are highly heterogeneous with cystic or hemorrhagic components and micro- or macroscopic lipids in 10% of cases [59,60]. Up to 10% of pheochromocytomas exhibit calcifications, usually punctate [45]. ACC, similarly, is highly heterogeneous, often featuring necrosis, hemorrhage, and calcifications. It shows predominantly peripheral enhancement after contrast administration. Other malignant lesions, such as neuroblastomas, appear as large, heterogeneous masses with calcifications, necrosis, and hemorrhage [7]. In contrast, regular margins are characteristic of benign lesions such as adenomas and myelolipomas. However, pheochromocytomas, despite their heterogeneity, also tend to have well-demarcated margins [61].

## 5. Suspicious MR Signs

MRI serves as a second-line imaging modality, especially when CT findings are inconclusive [62]. A standard MRI protocol for adrenal masses should include T1-weighted in-phase and opposed-phase dual-echo sequences, T2-weighted sequences (with and without fat suppression), and optionally, T1-weighted gadolinium-enhanced sequences [32].

Chemical shift imaging (CSI), which consists of T1-weighted in-phase (IP) and opposed-phase (OP) sequences, is the most critical parameter for characterizing adrenal lesions [63,64]. CSI detects microscopic intracellular fat through signal loss on opposed-phase sequences [7]. This signal loss can be assessed visually or quantitatively. Quantitative evaluation includes metrics such as the signal intensity index, where values > 16.5% or an adrenal-to-spleen ratio < 0.71 indicate adrenal adenomas [65]. Studies have shown that CSI has a sensitivity of 81–100% and a specificity of 94–100%, comparable to non-contrast CT [7,66]. However, CSI is less sensitive than washout CT for detecting lipid-poor adenomas, particularly when the unenhanced CT density exceeds 20 HU [67,68,69,70,71]. Notably, certain malignant lesions such as adrenocortical carcinoma, pheochromocytoma, and metastases from clear cell renal cell carcinoma may also exhibit signal loss on OP images, mimicking benign masses [72].

**Figure 11:**
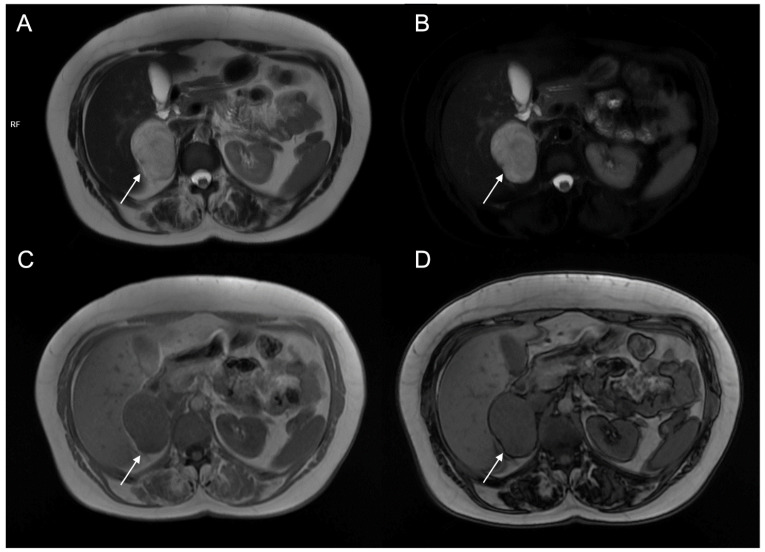
A 48-year-old female with hypertension and tachycardia. MRI revealed a solid mass (arrow) of the right adrenal gland measuring 55 × 42 mm. The mass was uniformly hyperintense on T2-weighted images (**A**) and on T2-weighted sequences with fat suppression (**B**), hypointense on T1-weighted in-phase image (**C**) without signal loss in the opposition-phase image (**D**). The clinical and radiological suspicion for pheochromocytoma was confirmed by histology.

T2-weighted sequences can identify the “light bulb sign,” characterized by uniformly high signal intensity, which is typical of pheochromocytomas (Figure 11). However, as Fonseca et al. noted, this sign occurs in less than 70% of pheochromocytomas due to the presence of hemorrhage, myxoid degeneration, or cystic components, which result in a more heterogeneous appearance. Additionally, necrotic areas in aggressive lesions, such as carcinomas or metastases, may cause false-positive light bulb signs [73].

A 2020 study by Wendy Tu et al. retrospectively analyzed 40 patients with adrenal metastases and 23 patients with lipid-poor adenomas at 1.5T and 3T MRI. They measured T2-weighted signal intensity (SI) ratios (SInodule/SIpsoas muscle), T2-weighted histogram features, and chemical shift SI indices. Their findings demonstrated that combining T2-weighted SI and T2-weighted heterogeneity improves the differentiation of metastases from lipid-poor adenomas [74].

Diffusion-weighted imaging (DWI) has been investigated for adrenal tumor characterization, but its diagnostic value has been limited due to significant overlap in apparent diffusion coefficient (ADC) values between benign and malignant adrenal lesions [71,75]. A study by Halefoglu et al. involving 108 patients with 126 adrenal masses found that CSI was more effective than DWI in differentiating adrenal metastases from lipid-poor adenomas [76]. Similarly, El-Kalioubie et al. examined 55 patients with 59 adrenal lesions and concluded that ADC values were not useful in distinguishing adrenal masses. Instead, lesion size and signal drop on OP sequences were stronger predictors of malignancy [77]. Although some studies suggest that primary adrenal carcinomas and lymphomas have higher ADC values than adenomas or metastases, these findings remain unconfirmed [60].

Contrast-enhanced MRI is valuable in cases of suspected pheochromocytomas due to their characteristic bright enhancement. However, it does not provide additional diagnostic information for adenomas. Becker-Weidman et al. studied 46 lipid-poor adrenal lesions with indeterminate CSI findings and found that combining T2-weighted sequences with contrast-enhanced T1-weighted three-dimensional gradient echo sequences accurately identified lipid-poor adenomas [78].

MRI is particularly useful for evaluating adrenal masses with unenhanced CT attenuation values between 10 and 30 HU. Lipid-poor malignant adrenal lesions, such as metastases and adrenocortical carcinoma (ACC), typically show low intensity on T1-weighted sequences without signal loss on OP sequences, moderate-to-high intensity on T2-weighted sequences, and lack a definitive washout pattern [7,58].

Future advancements may include the integration of proton magnetic resonance spectroscopy (MRS) for adrenal mass characterization. MRS offers the potential to assess the metabolic activity of adrenal lesions. A study by Dalavia CC et al. on 97 patients with adrenal nodules or masses demonstrated that combining the signal intensity index from CSI with MRS increased diagnostic accuracy for differentiating adrenal adenomas from non-adenomas [79].

As an aid in the characterisation of adrenal incidentalomas, we have summarised the main CT and MRI features suspected for malignancy in Table 1.

## 6. Future Directions: Radiomics

CT and MRI, while highly valuable, are not always sufficient to characterize all adrenal masses. Furthermore, biopsy is often limited due to associated risks and additional costs [80]. This has led to increased interest in emerging imaging techniques such as Radiomics, which aims to extract quantitative features from standard medical images that are not visible to the human eye. This process utilizes advanced computational methods to enhance the analysis and characterization of lesions [81,82,83,84]. Radiomics with texture analysis can reflect underlying tissue heterogeneity through the extraction of first-order features, describing the distribution of voxel intensity values, second-order features, reflecting the spatial relationship of voxel, and higher-order features [85,86,87].

In this context, several studies have evaluated the role of radiomics in differentiating between benign and malignant lesions and between their different subtypes, as shown in more detail below. However, as pointed out by Stanzione et al. in their systematic review, the application of radiomics in real life is currently limited by the poor and heterogeneous quality of radiomics studies in the literature. They found an absence of prospectively designed studies, poor data openness, and the lack of appropriate model validation while new efforts and improvements have been made for standardization of practice in radiomics. Moreover, another crucial challenge in radiomics reproducibility concerns the process of segmentation of the volume of interest. In this setting, automated segmentation of adrenal lesions would ideally represent the solution [88].

### 6.1. Texture Analysis in the Differentiation Between Benign and Malignant Lesions

One of the most important goals for texture analysis is to be a valid tool for characterising adrenal lesions differentiating benign from malignant lesions non-invasively, thus becoming a great support in the diagnostic-therapeutic workflow of these patients.

#### 6.1.1. Lipid-Poor Adenomas vs. Malignant Adrenal Nodules

In the case of finding indeterminate lesions with high density values on CT (>10 HU) and/or heterogeneous lesions, it is essential to distinguish lipid-poor adenomas (LPA) from malignant lesions. Therefore, several studies evaluated how texture analysis can provide an aid in differentiating lipid-poor adenomas from malignant lesions [89,90]. For example, the retrospective study by Ho et al. tried to evaluate how texture analysis with second-order features on unenhanced CT images, contrast-enhanced CT (CECT), and MRI was able to differentiate LPAs from other malignancies. They demonstrated that texture analysis showed a higher diagnostic performance for the diagnosis of malignancy, compared with CECT, in particular, through the second-order features’ long-run high grey-level emphasis, entropy, and short-run low grey-level emphasis [89]. The ability of texture analysis to differentiate these two entities derives from the intrinsic tissue heterogeneity of malignant lesions compared to LPAs, which are more homogeneous after contrast somministration. However, Ho et al. did not find substantial differences between texture analysis and unenhanced CT and MRI in discriminating LPAs from malignant lesions, because there were no statistically significant features able to distinguish the two entities. These results were justified by the fact that both LPA and malignancy on unenhanced CT and chemical shift MRI images were very heterogeneous, due to the uneven distribution of endo-lesional lipid zones in the former and the presence of necrosis in the latter [89].

Zhang et al., in their study conducted on unenhanced CT images of 292 patients with 141 adrenal LPAs and 151 non-adenomas lesions, created a nomogram model based on unenhanced CT that showed excellent diagnostic performance (AUC 0.96, sensitivity, 92.9%; specificity, 88.1%) in distinguishing LPA from other adrenal malignancies, suggesting how CECT could be potentially avoided in patients with confirmed diagnosis of LPA by texture analysis [90]. Similar results were obtained by Stanzione et al. in their study on 55 indeterminate adrenal lesions without fat signal drop on CSI at MR. Based on their results, they hypothesised that radiomics and machine learning could be a problem-solving tool to characterise indeterminate adrenal lesions as benign or malignant, avoiding unnecessary and expensive CECT or PET/CT and invasive diagnostic tools such as biopsy [91].

#### 6.1.2. Lipid-Poor Adenomas vs. Pheochromocytoma

**Figure 12:**
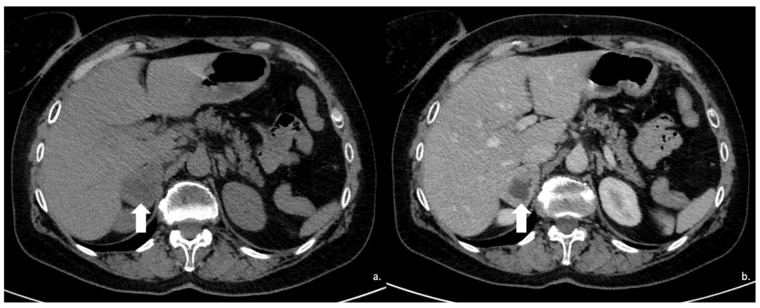
(**a**) Unenhanced and (**b**) venous CT phases show a solid lesion (arrow) in the right adrenal gland with a heterogeneous contrast-enhancement due to necrotic-colliquative phenomena; on histological examination, the lesion was a pheochromocytoma.

**Figure 13:**
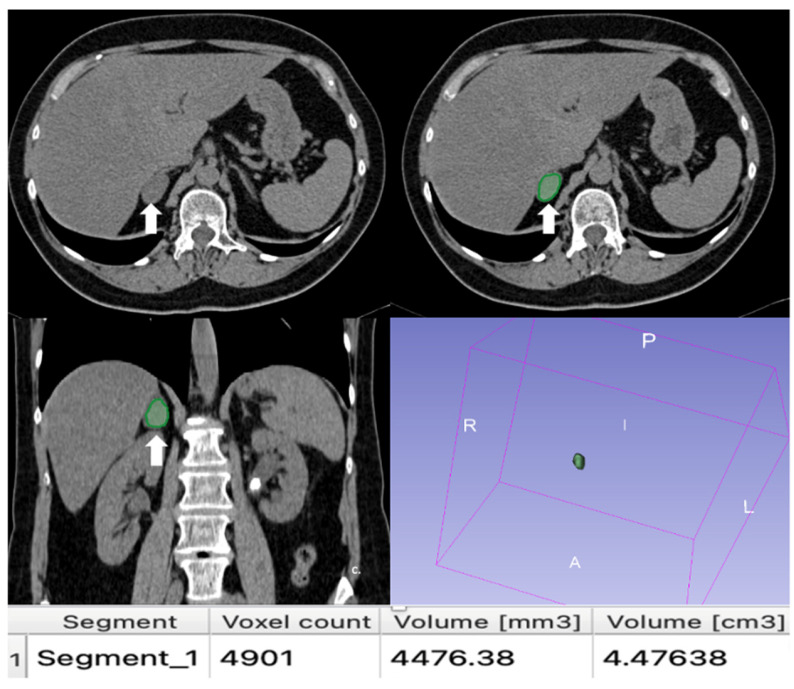
An example of segmentation of an LPA in the right adrenal gland (arrow) in an unenhanced CT phase using 3D Slicer software (version 4.11). The volume of interest (VOI) of the LPA was manually drawn covering the whole lesion.

Pheochromocytoma is a rare adrenal tumour, the prevalence of which is estimated to be 0.1–0.6% in the hypertensive adult population. The incidence in the general population is around 0.05% [92]. In 5–10% of cases it is associated with diseases such as multiple endocrine neoplasia type II (MEN II, both IIa and IIb), or other conditions such as von Hippen Lindau disease [93]. Its clinical manifestations are uncontrollable secondary hypertension, cardiac dysfunction, and neurological alterations such as haemicrania [92]. On CT they appear as large, heterogeneous masses with necrosis (Figure 12). After contrast medium they tend to enhance avidly in the arterial phase with a slow washout. However, sometimes they may show a rapid washout, similar to adrenal adenoma [94]. Moreover, in 10–20% of cases pheochromocytomas show lipid content with densitometric values < 10 HU. Therefore, because of the possible overlap with adenomas, several studies evaluated if texture analysis can differentiate subclinical pheochromocytomas (sPHEO) from lipid-poor adenomas (Figure 13). Liu et al. conducted a study involving 183 patients with LPA and 86 patients with sPHEO on unenhanced CT images [95]. They developed prediction models and scoring systems for distinguishing sPHEO from LPA on non-contrast CT, suggesting the potential of using a non-invasive imaging method to differentiate and predict the histology of adrenal lesions. Another work by Yi et al. demonstrated the potential of radiomics and textural features for distinguishing sPHEO from LPA [96]. Also, Kong et al. developed a radiomics model combining the radiomics signature and symptoms for predicting the presence of PHEO, against other adrenal lesions, before initial treatment [97].

#### 6.1.3. Benign Adrenal Lesions vs. Metastasis

**Figure 14:**
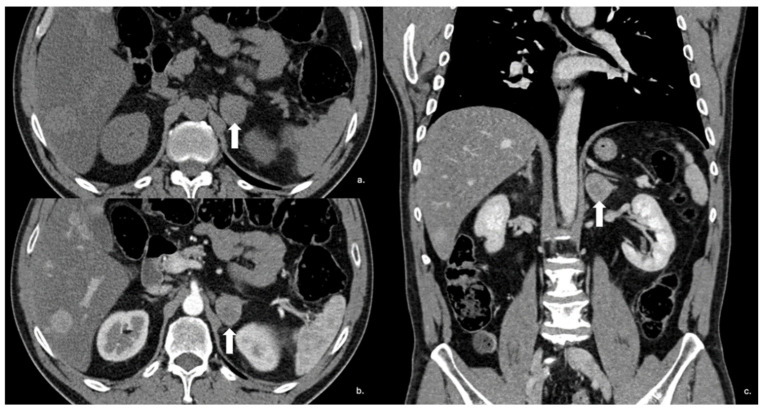
Axial unenhanced (**a**), arterial (**b**), and coronal venous (**c**) CT phases show a lung carcinoid metastasis (arrow) of the left adrenal gland. Additional hypervascular metastasis can be seen in the liver, particularly in the arterial phase.

Although adenomas are the most frequently detected adrenal lesions, the risk of malignancy increases significantly in patients with an underlying tumour disease [29] (Figure 14). O’Shea et al. analysed 86 patients with confirmed biopsy for adrenal metastasis and 55 patients with LPA who underwent contrast-enhanced, portal-venous phase CT of the abdomen [98]. They demonstrated that the nomogram through the association of clinic (age and history of known malignancy) with first and higher-order radiomic features achieves an area under the curve (AUC) of 97.2% in the training cohort and 90.4% in the validation cohort, emphasising the ability to differentiate metastatic from benign lesions. Similar results were also obtained by Shi et al. on 265 histologically confirmed adrenal masses of which 101 were metastases, 98 were pheochromocytomas, and 66 were LPAs [99].

Winkelmann et al. also carried out a study using the application of radiomics on dual-energy CT imaging. Radiomics was able to differentiate between adrenal adenomas and metastases. However, according to their study, dual-energy CT post-processing was more accurate than radiomics through virtual non-contrast images, iodine quantification, and fat fraction analysis [100].

**Figure 15:**
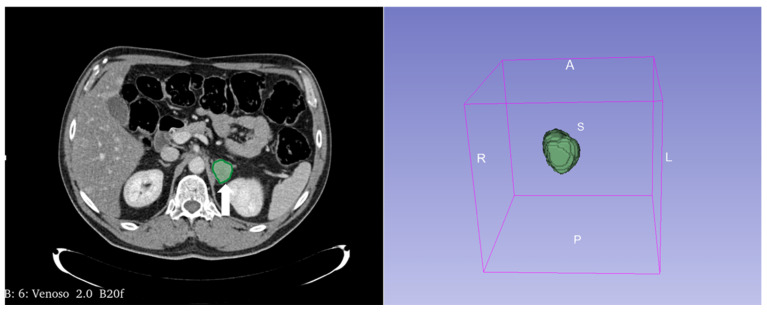
Segmentation of the metastatic lesion (arrow) of the left adrenal gland (same patient of Figure 4) on venous phase CT, using 3D-Slicer Software, version 4.11.

More specifically, the study by Andersen et al. focused on adrenal metastases from lung cancer [101]. They found several texture parameters statistically significantly in differentiating metastatic and benign adrenal lesions in patients with lung cancer (Figure 15).

#### 6.1.4. Adenoma vs. Adrenal Carcinoma

**Figure 16:**
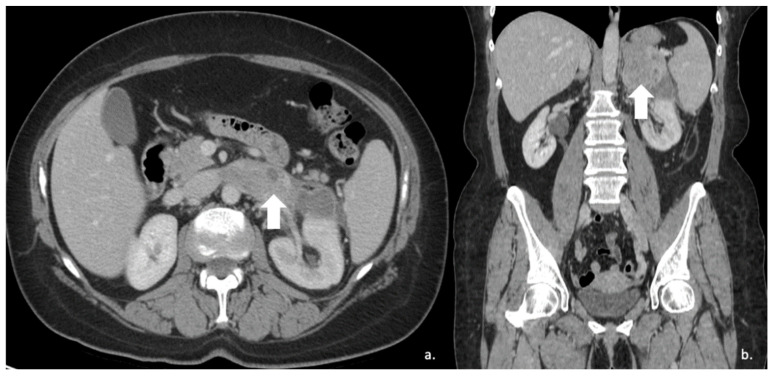
(**a**) Axial and (**b**) coronal venous phases CT show an ACC of the left adrenal gland presenting as a large mass (arrow) of heterogeneous appearance with extensive neoplastic thrombosis of the ipsilateral renal vein.

Primary ACC account for 0.02% of all malignancies. On imaging they often appear as masses with dimensions > 4 cm, necrosis, hemorrhage, and HU > 20 on CT imaging (Figure 16) [102]. Given the different therapeutic approach of these tumour lesions, a correct characterization is crucial. The study by Elmohr et al. demonstrates a positive correlation between the heterogeneity of a lesion and the risk of carcinoma [103]. ACC showed an increased attenuation, represented by grey level and heterogeneity compared to adenoma, which appear to be more homogeneous in texture. In fact, carcinoma tend to be more heterogenous on CECT, because of the presence of necrosis, haemorrhage, and calcifications. The study by Torresan et al., confirmed the results, extracting several parameters among first- and second-order features with high diagnostic accuracy (sensitivity and specificity over 90%) in discriminating between benign lesions and ACC [3].

### 6.2. Texture Analysis in the Differentiation of Malignant Lesions

Radiomics could be not only a valid tool to differentiate tumour lesions, but it has also been evaluated as a prognostic tool that could be useful in the diagnostic and therapeutic work-up of these tumours. Indeed, studies such as that of Ahmed et al. have been conducted in order to predict preoperatively the value of Ki-67 expression in ACC through CECT-derived radiomic features [104]. Ki-67 is a protein used as a marker of cellular proliferation for tumours, including ACC, and is considered one of the most important prognostic markers for local recurrence of ACC [105]. It can be quantified only through histopathological examination of resected tumour tissue. Therefore, an early assessment of its value in ACC would be of great importance [105]. They found out that a radiomic signature derived from CECT could be in the future a non-invasive predictor of Ki-67 expression status in patients with ACC. Moreover, in the case of pheochromocytomas, it is important to have information on the secretory or histopathological properties of aggressiveness. Indeed, these tumours can be characterised by a low or high histopathological aggressiveness as well as by a more pronounced hormone-secreting activity. The study by De Leo et al. therefore confirmed texture analysis as a valid tool on contrast-enhanced CT images as a non-invasive, quantitative tool for helping in the characterisation of the clinical, biochemical, and histopathological features of PHEO [106].

## 7. Conclusions

Both CT and MR have a pivotal role in adrenal imaging. When an adrenal incidentaloma is identified, the main purpose of imaging should be to try to distinguish between benign and malignant lesion. Therefore, the radiologist must be able to detect features of malignancy by paying attention to parameters such as size, basal density value on CT, the growth or the stability of the lesion over time, the absolute/relative washout, and signal intensity on MRI. Moreover, radiomics is a valuable aid in cases where CT and MRI cannot characterize adrenal masses and could be an important prognostic tool in the future.

## Figures and Tables

**Table 1:** Diagnostic imaging features suspicions of malignancy.

Criteria Favoring a Malignant Mass	Explanation
Absence of macroscopic fat	>20 HU on unenhanced CT; No signal loss on T1-weighted opposed-phase sequences.
APW ^1^ < 60% and RPW ^2^ < 40%	Rapid enhancement and slow washout on contrast imaging raise the suspicion of malignant.
Large size	AI ^3^ > 4 cm has an increased likelihood of being malignant.
Growth	Lesions that increase in size are more suspicious for malignancy than those that remain stable for over a year.
Heterogeneous appearance	AI ^1^ that shows necrosis, haemorrhage and calcifications has a higher probability of being malignant.
Irregular margins	Irregular margins can indicate an increased probability of malignancy

^1^ Absolute percentage washout. ^2^ Relative percentage washout. ^3^ Adrenal incidentaloma.

## Data Availability

All images included in the review are the property of the authors.

## Abbreviations

The following abbreviations are used in this manuscript:

AI	adrenal incidentaloma
CT	Computed Tomography
MR	Magnetic Resonance
LPA	lipid-poor adenoma
LRA	lipid-rich adenoma
ACC	adrenocortical carcinoma
FDG-PET	Fluorine-18-2-fluoro-2-deoxy-Dglucose-positron emission tomography
HU	Hounsfield unit
ROI	region of interest
APW	absolute percentage washout
RPW	relative percentage washout
CSI	Chemical Shift Imaging
DWI	Diffusion-weighted imaging
ADC	apparent diffusion coefficient
PHE	Pheochromocytoma
